# Real-Time PPP-RTK Performance Analysis Using Ionospheric Corrections from Multi-Scale Network Configurations

**DOI:** 10.3390/s20113012

**Published:** 2020-05-26

**Authors:** Dimitrios Psychas, Sandra Verhagen

**Affiliations:** 1 Department of Geoscience and Remote Sensing, Delft University of Technology, PO Box 5048, 2600 GA Delft, The Netherlands; Sandra.Verhagen@tudelft.nl; 2 Fugro Innovation & Technology B.V., PO Box 130, 2630 AC Nootdorp, The Netherlands

**Keywords:** GNSS, PPP-RTK network and user, integer ambiguity resolution (IAR), ionospheric corrections, network density, convergence time

## Abstract

The long convergence time required to achieve high-precision position solutions with integer ambiguity resolution-enabled precise point positioning (PPP-RTK) is driven by the presence of ionospheric delays. When precise real-time ionospheric information is available and properly applied, it can strengthen the underlying model and substantially reduce the time required to achieve centimeter-level accuracy. In this study, we present and analyze the real-time PPP-RTK user performance using ionospheric corrections from multi-scale regional networks during a day with medium ionospheric disturbance. It is the goal of this contribution to measure the impact the network dimension has on the ambiguity-resolved user position through the predicted ionospheric corrections. The user-specific undifferenced ionospheric corrections are computed at the network side, along with the satellite phase biases needed for single-receiver ambiguity resolution, using the best linear unbiased predictor. Such corrections necessitate the parameterization of an estimable user receiver code bias, on which emphasis is given in this study. To this end, we process GPS dual-frequency data from four four-station evenly distributed CORS networks in the United States with varying station spacings in order to evaluate if and to what extent the ionospheric corrections from multi-scale networks can improve the user convergence times. Based on a large number of samples, our experimental results showed that sub-10 cm horizontal accuracy can be achieved almost instantaneously in the ionosphere-weighted partially-ambiguity-fixed kinematic PPP-RTK solutions based on corrections from a network with 68 km spacing. Most of the solutions (90%) were shown to require less than 6.0 min, compared to the ionosphere-float PPP solutions that needed 68.5 min. In case of sparser networks with 115, 174 and 237 km spacing, 50% of the horizontal positioning errors are shown to become less than one decimeter after 1.5, 4.0 and 7.0 min, respectively, while 90% of them require 10.5, 16.5 and 20.0 min. We also numerically demonstrated that the user’s convergence times bear a linear relationship with the network density and get shorter as the density increases, for both full and partial ambiguity resolution.

## 1. Introduction

PPP-RTK is the realization of integer ambiguity resolution-enabled precise point positioning (PPP), which was first conceptualized by Wübbena et al. [1]. The development of the PPP technique [2] enabled single-receiver users to achieve positioning accuracy on the order of a few centimeters and of a few decimeters in static and kinematic mode, respectively, using precise satellite orbits and clocks [3,4].

In the frame of standard PPP, such accuracy can be obtained using data over long observational spans, ranging from tens of minutes to several hours [5,6]. This has its roots in the incapability to resolve the phase ambiguities to integers since they cannot be separated from the receiver and satellite hardware biases existing in the code and phase data. To this end, PPP-RTK extends the PPP technique by means of providing single-receiver users, next to orbits and clocks, information about the satellite phase and code biases. This information, when properly provided, allows to recover the integerness of user-ambiguities and thus to enable single-receiver integer ambiguity resolution (IAR) [7,8,9,10,11,12].

It has to be remembered, though, that IAR is not the goal in itself. The purpose of resolving the carrier-phase ambiguities to their integers is to reduce the convergence time of PPP solutions, which is mainly governed by the presence of these unknown ambiguities. To do so, one has to map the data-driven ambiguities to their *correct* integers successfully. The reliability of this process heavily depends on the underlying model strength, which is inextricably linked to the number of available observations and unknown parameters, the stochastic model, the receiver-satellite geometry and the atmospheric errors’ modeling. It has been shown that reliable ambiguity fixing in single-system PPP-RTK can be achieved when data over multiple epochs are accumulated, ranging from 30 to 60 min [13].

Such a long time span is not attractive, of course, for critical real-time applications that require fast high-precision positioning. One of the major bottlenecks of fast IAR is the presence of ionospheric delays, which need to be explicitly parameterized for in an uncombined GNSS formulation, where no a priori differencing or inter-frequency combinations take place. Such a model is weak in terms of its ambiguity resolution capabilities, due to the increased number of unknown parameters that need to be estimated.

However, an uncombined GNSS formulation has several advantages and flexibility that have already been identified [14,15,16,17]. Such an approach allows one to use the original code and phase data, usually uncorrelated, and keeps all parameters in the observation equations, thus allowing not only a flexible and rigorous extension to multi-GNSS and/or multi-frequency applications but also a possible further model strengthening. The latter can be achieved if one increases the number of observations by employing, for instance, a priori information for the ionosphere.

Successful ambiguity resolution, and thus convergence time, can be achieved much faster when precise a priori ionospheric information is provided to the users. In the present contribution, we make use of the uncombined GPS dual-frequency model to determine network-derived estimable satellite phase biases and predicted ionospheric corrections for single-receiver *fast* IAR. Although multi-frequency and multi-GNSS PPP-RTK have proven to bring an improvement in the convergence time [18,19], the focus has been given mostly to the ionosphere-float models. In this study we focus solely on the ionosphere-weighted model.

During recent years, there have been a few studies investigating the use of ionospheric information to reduce the PPP-RTK convergence times. Teunissen et al. [11] demonstrated that single-epoch ionosphere-weighted PPP-RTK can achieve mm-level horizontal accuracy using corrections from two small-scale networks with inter-station distances of around 27 and 60 km in different locations. Using a different parameter mapping and two networks with inter-station distances ranging from 60 to 100 km in different locations, Zhang et al. [20] showed a comparable single-epoch PPP-RTK performance. Although an excellent user performance was demonstrated in both studies, they were both based on a single-epoch model which is not always strong enough to achieve successful IAR and thus high accuracy, especially in sparser networks when ionospheric residuals are present. Li et al. [21] showed that instantaneous IAR at the user level is possible by using linearly interpolated atmospheric corrections from a regional network of 60 km spacing. Although cm-level accuracy was demonstrated based on a 2-h dataset, the generation of atmospheric corrections would be successful only when the ambiguities of the reference stations were fixed. A similar performance was found in Li et al. [22]. In both studies, though, the necessity to parameterize for the estimable user receiver code bias was not discussed, as it was assumed that the receiver clock offset can absorb it. Banville et al. [6] demonstrated that sub-decimeter positioning accuracy can be achieved instantaneously for 21 out of 24 h solutions in a day with quiet ionospheric conditions, by using regional corrections from a 150 km network with minimum distance to the user of 97 km. Based on three 1-h time intervals and ionospheric predictions from two unevenly distributed networks in different locations with the largest inter-station distances equal to 580 and 295 km, respectively, Wang et al. [23] found that 10 s are required to let most of the horizontal positioning errors to converge to less than 10 cm.

Therefore, although there has been given attention to the role the ionospheric corrections can play in reducing the PPP-RTK convergence time, the impact of the network dimension on the user’s performance has not been explored in detail and needs further attention. Moreover, a rigorous assessment of the ionosphere-weighted PPP-RTK user performance has not yet been presented in the existing literature, which has been restricted to a small number of samples, unlike for this of the ionosphere-float model [13,18,24].

The goal of this contribution is to systematically analyze the performance of real-time ionosphere-weighted PPP-RTK by means of analyzing a large number of user ambiguity-resolved position solutions based on ionospheric corrections from regional networks of varying station spacing in the same area and in a day with existing ionospheric disturbance. Moreover, our aim is to provide numerical insight into what extent the ionospheric information can reduce the convergence times based on the network density and to show the capabilities of a sparse network in providing fast high-precision GNSS parameter estimation. To that end, we use the best linear unbiased predictor (BLUP) to interpolate undifferenced ionospheric corrections within the network processing. We also emphasize on the correct interpretation of the estimable ionospheric corrections, which is essential so as to estimate the corresponding biases at the user side.

This contribution is organized as follows. Section 2 presents the underlying model and estimable parameters of both PPP-RTK network and user components. Further, we review the predictor for generating user-specific ionospheric corrections based on network-derived information. In Section 3 the data, setup and processing strategies are described, along with an analysis of the relevant estimated network corrections. Following this, we present and analyze the PPP-RTK user performance results based on a large number of solutions. We conclude in Section 4.

## 2. Methodology

In this section, we first present the PPP-RTK network and user observation models based on uncombined measurements, and then we review a strategy for the prediction of user-specific ionospheric corrections based on network-derived information.

### 2.1. GNSS Observation Equations

Let us commence with the set of uncombined carrier-phase and pseudorange observation equations. By uncombined observations, we mean that no inter-frequency linear combinations or differencing are applied in the observation domain, in order to apply dynamic constraints on all parameters. We dispense with the term undifferenced since an uncombined observation per definition is undifferenced. For a receiver-satellite combination r−s at frequency *j* and at a single epoch, the uncombined phase ($\phi_{r,j}^{s}$) and code ($p_{r,j}^{s}$) measurements are defined as [25,26]:

(1) $$\begin{array}{cc} E(\phi_{r,j}^{s}) & =\rho_{r}^{s}+\left(dt_{r}-dt^{s}\right)+m_{r}^{s}\tau_{r}-\mu_{j}\iota_{r}^{s}+\lambda_{j}\left(\delta_{r,j}-\delta_{,j}^{s}+a_{r,j}^{s}\right)\\ E(p_{r,j}^{s}) & =\rho_{r}^{s}+\left(dt_{r}-dt^{s}\right)+m_{r}^{s}\tau_{r}+\mu_{j}\iota_{r}^{s}+\left(d_{r,j}-d_{,j}^{s}\right) \end{array}$$

where $\rho_{r}^{s}$ denotes the receiver-satellite geometric range. The symbols $dt_{r}$ and $dt^{s}$ denote the receiver and satellite clock parameters, respectively. $\tau_{r}$ represents the wet component of the zenith tropospheric delay (ZTD), since the hydrostatic counterpart can be a priori corrected, and $m_{r}^{s}$ is the tropospheric mapping function. The first-order slant ionospheric delays experienced on the first frequency (L1) are denoted by $\iota_{r}^{s}$ and are linked to the observations through the frequency-dependent ionospheric coefficient $\mu_{j}$. The frequency-dependent receiver and satellite phase biases are denoted by $\delta_{r,j}$ and $\delta_{,j}^{s}$, respectively, while $d_{r,j}$ and $d_{,j}^{s}$ represent the receiver and satellite code biases. The integer phase ambiguity is denoted by $a_{r,j}^{s}$ and is linked to the phase data through the wavelength at frequency *j*, $\lambda_{j}$. E(·) denotes the expectation operator.

The above variables have a receiver index r=1,…,n with *n* being the number of receivers, a frequency index j=1,…,f with *f* being the number of frequencies (f=2 in this study), and a satellite index s=1,…,m with *m* being the number of tracked satellites. The code biases, phase biases and integer ambiguities are assumed to be time-invariant, unless a cycle slip occurs for the latter, while the other variables are time-dependent. All quantities are expressed in units of range, apart from the phase biases and ambiguities that are expressed in cycles. The ionospheric coefficient is defined as the squared ratio of frequencies: $\mu_{j}={\left(f_{1}/f_{j}\right)}^{2}$.

The above observation equations apply for both PPP-RTK components: user and network. Moreover, the receiver positions are assumed to be a priori known in the network component, allowing one to subtract $\rho_{r}^{s}$ from the observations, given that precise orbits have been used. This is not the case for the user, as the observation equations need to be linearized with respect to the unknown user receiver position.

### 2.2. PPP-RTK Network

The uncombined formulation of Equation (1) cannot be used directly to estimate all the unknown parameters as is, i.e., in an unbiased form, since the system of observation equations is rank-deficient. According to S-system theory [27,28], to solve for these rank deficiencies and obtain a full-rank network system, one has to find linearly independent estimable functions of these parameters based on a minimum constraint set or S-basis, the number of which equals to the rank deficiency. In this contribution, we opt for a *Common Clocks* S-system [29] resulting in a reformulation of Equation (1) into:

(2) $$\begin{array}{cc} E(\Delta \phi_{r,j}^{s}) & =d{\tilde{t}}_{r}-d{\tilde{t}}^{s}+m_{r}^{s}\tau_{r}-\mu_{j}{\tilde{\iota }}_{r}^{s}+\lambda_{j}\left({\tilde{\delta }}_{r,j}-{\tilde{\delta }}_{,j}^{s}+{\tilde{a}}_{r,j}^{s}\right)\\ E(\Delta p_{r,j}^{s}) & =d{\tilde{t}}_{r}-d{\tilde{t}}^{s}+m_{r}^{s}\tau_{r}+\mu_{j}{\tilde{\iota }}_{r}^{s} \end{array}$$

where the interpretation of the estimable parameters (denoted using the tilde $\tilde{\left(\cdot \right)}$ symbol) and the S-basis parameters are listed in Table 1. The terms $\Delta \phi_{r,j}^{s}$ and $\Delta p_{r,j}^{s}$ denote the observed-minus-computed phase and code measurements, respectively, which include the receiver and satellite positions.

From Table 1, one can observe that all estimable parameters are functions of their original counterparts, biased by the S-basis parameters, except for the ZTD. This model is based on the ionosphere-float formulation, where the slant delays are estimated as unknown parameters for every receiver-satellite combination.

### 2.3. Prediction of Ionospheric Corrections

Given the PPP-RTK network-derived estimates and the fact that the ionosphere decorrelates with increasing inter-station distance [30], the slant ionospheric delays of the network stations can serve as the basis for providing an educated guess of the user-specific delays. Therefore, in the present contribution, we treat the undifferenced (biased) slant ionospheric delay estimates of the reference network stations as observable random signals to spatially predict the unobservable random ionospheric signals at the user side, per satellite and per epoch, based on the spatial coherence of ionosphere. Several interpolation methods have been proposed for this matter, and it has been shown that their performance is comparable [31]. In this study, we apply the least-squares prediction theory [32] and use the trend-signal-noise model, which forms the basis of the least-squares collocation method [33].

**Figure 1:**
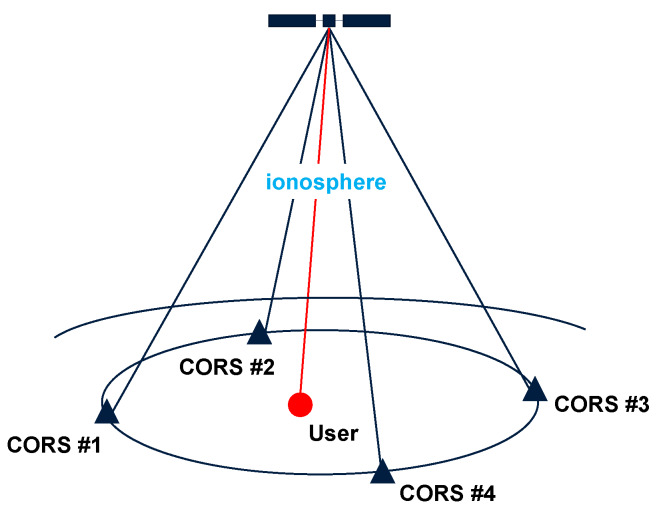
Schematic principle of the satellite-by-satellite approach used in predicting user-specific slant ionospheric corrections per satellite and per epoch.

Based on the satellite-by-satellite approach, we assume that the ionospheric delay experienced between the user and a certain satellite can be represented by the mean value of the delays experienced between the network receivers and the same satellite within the measurement cone, depicted in Figure 1, the base of which is formed by the network receiver positions and its vertex from the satellite position. We consider this assumption to be valid in the case of local and regional networks and in the absence of high ionospheric activity. Let us therefore consider the partitioned linear system of equations that relates the vector $\hat{\tilde{\iota }}$ of observable slant delays of the reference stations, where $\hat{\tilde{\iota }}={\left[{\hat{\tilde{\iota }}}_{1}^{T},\ldots ,{\hat{\tilde{\iota }}}_{n}^{T}\right]}^{T}$ and ${\hat{\tilde{\iota }}}_{r}={\left[{\hat{\tilde{\iota }}}_{r}^{1},\ldots ,{\hat{\tilde{\iota }}}_{r}^{m}\right]}^{T}$, with the vector $\bar{\iota }={\left[{\bar{\iota }}^{1},\ldots ,{\bar{\iota }}^{m}\right]}^{T}$ of the spatial mean ionospheric delays per satellite and the unobservable vector ${\tilde{\iota }}_{net\to u}={\left[{\tilde{\iota }}_{net\to u}^{1},\ldots ,{\tilde{\iota }}_{net\to u}^{m}\right]}^{T}$ that contains the user-predicted ionospheric delays:

(3) $$E\left(\left[\begin{array}{c} \hat{\tilde{\iota }}\\ {\tilde{\iota }}_{net\to u} \end{array}\right]\right)=\left[\begin{array}{cc} e_{n}⊗I_{m} & C_{n}⊗e_{m}\\ I_{m} & 0_{m\times (n-1)} \end{array}\right]\left[\begin{array}{c} \bar{\iota }\\ {\tilde{d}}_{p^{\prime }r,\mathrm{GF}} \end{array}\right]$$

where *m* and *n* denote the number of satellites and network stations, $I_{n}$ denotes a unit matrix of order *n*, $e_{n}$ is an *n*-vector having ones as its entries, $C_{n}$ denotes a unit matrix of order *n* with its first column removed, and ${\tilde{d}}_{p^{\prime }r,\mathrm{GF}}={\left[{\tilde{d}}_{p^{\prime }2,\mathrm{GF}},\ldots ,{\tilde{d}}_{p^{\prime }n,\mathrm{GF}}\right]}^{T}$. ⊗ denotes the Kronecker product.

In this model, we parameterized the trend in terms of the satellite-wise mean ionospheric delay and the network receiver differential code biases (DCBs). This was done for receiver code bias calibration reasons, considering as S-basis the receiver DCB of one of the network receivers contributing to the spatial prediction. It has to be mentioned at this point that the pivot receivers in the PPP-RTK network processing and the ionospheric delay prediction did not have to be necessarily the same. In the current section, we assume that the pivot receiver is the first one for notational convenience.

The variance-covariance (vc-) matrix of the observable ionospheric signals captures both the measurement and the signal noise. Since the network processing is assumed to continuously generate PPP-RTK corrections, the network-derived slant ionospheric delay estimates gain a high precision over time such that their corresponding vc-matrix can be neglected. In such a case, we are only left with the vc-matrix of the signals. To model the between-receiver spatial correlation of the ionosphere, we use a Gaussian function as it is a decreasing autocorrelation function that guarantees the positive definiteness of the ionospheric vc-matrix [34]:

(4) $$h_{ij}=c_{\iota }^{2}\exp\left(-{\left(\frac{l_{ij}}{l_{0}}\right)}^{2}\right)$$

where $h_{ij}$ is the correlation function for receivers *i* and *j*, $l_{ij}$ is the distance between them, and $l_{0}$ is a pre-defined applicable inter-station distance for ionospheric signal spatial correlation. The variance $c_{\iota }^{2}$ denotes the value of the covariance function when the inter-station distance is zero. exp(·) denotes the natural exponential function. Based on all of the above, the ionospheric signal vc-matrix is defined as:

$$D\left(\left[\begin{array}{c} \hat{\tilde{\iota }}\\ {\tilde{\iota }}_{net\to u} \end{array}\right]\right)=\left[\begin{array}{cc} H_{ij} & H_{iu}\\ H_{iu}^{T} & H_{uu} \end{array}\right]⊗I_{m},\;\;i,j=1,\ldots ,n$$

where D(·) denotes the dispersion operator and *H* denotes the correlation matrix, based on Equation (4).

Therefore, based on BLUP [32,35], the stochastic user-specific ionospheric corrections read as:

(5) $${\hat{\tilde{\iota }}}_{net\to u}=\hat{\bar{\iota }}+\left[\left(H_{iu}^{T}H_{ij}^{-1}\right)⊗I_{m}\right]\left(\hat{\tilde{\iota }}-\left[e_{n}⊗I_{m}\right]\;\hat{\bar{\iota }}-\left[C_{n}⊗e_{m}\right]\;{\hat{\tilde{d}}}_{p^{\prime }r,\mathrm{GF}}\right)$$

with the best linear unbiased estimators of $\bar{\iota }$ and ${\tilde{d}}_{p^{\prime }r,\mathrm{GF}}$ being obtained from the normal equation:

(6) $$\left[\begin{array}{cc} \left(e_{n}^{T}H_{ij}^{-1}e_{n}\right)⊗I_{m} & \left(e_{n}^{T}H_{ij}^{-1}C_{n}\right)⊗e_{m}\\ \left(C_{n}^{T}H_{ij}^{-1}e_{n}\right)⊗e_{m}^{T} & (C_{n}^{T}H_{ij}^{-1}C_{n})\cdot m \end{array}\right]\left[\begin{array}{c} \hat{\bar{\iota }}\\ {\hat{\tilde{d}}}_{p^{\prime }r,\mathrm{GF}} \end{array}\right]=\left[\begin{array}{c} \left(e_{n}^{T}H_{ij}^{-1}\right)⊗I_{m}\\ \left(C_{n}^{T}H_{ij}^{-1}\right)⊗e_{m}^{T} \end{array}\right]\hat{\tilde{\iota }}$$

For the expectation of the user-predicted ionospheric corrections, we have:

(7) $$E\left({\hat{\tilde{\iota }}}_{net\to u}^{\;s}\right)={\tilde{\iota }}_{net\to u}^{\;s}=\iota_{u}^{s}-{\tilde{d}}_{,\mathrm{GF}}^{s},\;\mathrm{with}\;{\tilde{d}}_{,\mathrm{GF}}^{s}=d_{,\mathrm{GF}}^{s}-d_{p^{\prime },\mathrm{GF}}$$

Therefore, the predicted ionospheric delays are biased, apart from the satellite DCBs, by the network pivot receiver DCB. This needs to be carefully considered when applied at the user model and will be discussed in the next section. The predictor variance is computed with the variance propagation law.

### 2.4. PPP-RTK User

This section presents the ionosphere-float and ionosphere-weighted variants of the user’s model.

#### 2.4.1. Ionosphere-Float Model

The network-derived corrections that enable the PPP-RTK realization are the estimable satellite clocks and satellite phase biases. If we linearize the observation equations, shown in Equation (1), with respect to the unknown user position (index *r* changes to *u*) and apply both satellite orbits and network-derived corrections, the user’s single-system dual-frequency uncombined phase and code observation equations turn into:

(8) $$\begin{array}{cc} E(\Delta \phi_{u,j}^{s}+{\hat{\tilde{dt}}}^{s}+\lambda_{j}{\hat{\tilde{\delta }}}_{,j}^{s}) & =E\left(\Delta {\tilde{\phi }}_{u,j}^{s}\right)=g_{u}^{s^{T}}\Delta x_{u}+d{\tilde{t}}_{u}+m_{u}^{s}\tau_{u}-\mu_{j}{\tilde{\iota }}_{u}^{s}+\lambda_{j}\left({\tilde{\delta }}_{u,j}+{\tilde{a}}_{u,j}^{s}\right)\\ E(\Delta p_{u,j}^{s}+{\hat{\tilde{dt}}}^{s}) & =E\left(\Delta {\tilde{p}}_{u,j}^{s}\right)=g_{u}^{s^{T}}\Delta x_{u}+d{\tilde{t}}_{u}+m_{u}^{s}\tau_{u}+\mu_{j}{\tilde{\iota }}_{u}^{s} \end{array}$$

where $\Delta x_{u}$ denotes the user position increment vector and $g_{u}^{s}$ denotes the 3-vector containing the line-of-sight unit vectors. The precise satellite orbits are assumed to be included in the observed-minus-computed terms. In the case that both network and user models employ the same S-system, the parameter estimability and interpretation between them remains invariant.

From Table 1, one is able to recognize that the user’s receiver phase biases and integer ambiguities are not linearly dependent anymore since the integer ambiguities of the pivot satellite are taken as S-basis in this contribution, making the two parameters separable. The user’s ambiguities are now of double-differenced form and, therefore, integer-estimable.

The stochastic model, which is captured by the vc-matrix of the uncombined phase and code measurements, of the single-epoch single-system ionosphere-float model is given as:

(9) $$Q_{yy}=\mathrm{blkdiag}\left(Q_{\Delta {\tilde{\phi }}_{u}\Delta {\tilde{\phi }}_{u}},Q_{\Delta {\tilde{p}}_{u}\Delta {\tilde{p}}_{u}}\right),\;\;\mathrm{with}\;\;Q_{⋄⋄}=C_{⋄⋄}⊗W_{u}^{-1},\;\;\mathrm{and}\;\;⋄\in \left\{\Delta {\tilde{\phi }}_{u},\Delta {\tilde{p}}_{u}\right\}$$

where $y={\left[\Delta {\tilde{\phi }}_{u}^{T},\Delta {\tilde{p}}_{u}^{T}\right]}^{T}$ denotes the complete 4m measurement vector with $\Delta {\tilde{\phi }}_{u}\;=\;{\left[\Delta {\tilde{\phi }}_{u,1}^{1},\ldots ,\Delta {\tilde{\phi }}_{u,1}^{m},\Delta {\tilde{\phi }}_{u,2}^{1},\ldots ,\Delta {\tilde{\phi }}_{u,2}^{m}\right]}^{T}$ and $\Delta {\tilde{p}}_{u}={\left[\Delta {\tilde{p}}_{u,1}^{1},\ldots ,\Delta {\tilde{p}}_{u,1}^{m},\Delta {\tilde{p}}_{u,2}^{1},\ldots ,\Delta {\tilde{p}}_{u,2}^{m}\right]}^{T}$, and the frequency-specific zenith-referenced standard deviations of the phase and code data are captured in the sub-matrices $C_{\Delta {\tilde{\phi }}_{u}\Delta {\tilde{\phi }}_{u}}=\mathrm{diag}\left(\sigma_{\phi_{u,1}}^{2},\sigma_{\phi_{u,2}}^{2}\right)$ and $C_{\Delta {\tilde{p}}_{u}\Delta {\tilde{p}}_{u}}=\mathrm{diag}\left(\sigma_{p_{u,1}}^{2},\sigma_{p_{u,2}}^{2}\right)$, respectively. The matrix $W_{u}=\mathrm{diag}\left(w_{u}^{1},\ldots ,w_{u}^{m}\right)$ contains the satellite elevation-dependent weights $w_{u}^{s}=\sin^{2}\left(\beta_{u}^{s}\right)$ of the GNSS measurements, with $\beta_{u}^{s}$ denoting the elevation of satellite *s* from receiver *u*. The notations diag and blkdiag denote a diagonal and a block diagonal matrix, respectively.

The user model consisting of Equations (8) and (9) is the so-called ionosphere-float model, in which the biased slant ionospheric delays are estimated as unknown parameters. As a result, data over a long observational time span need to be accumulated for the position solution to gain high precision.

#### 2.4.2. Ionosphere-Weighted Model

If precise ionospheric information is available, the user’s model will be strengthened, which will improve the ambiguity resolution performance and, therefore, shorten the convergence time. The ionospheric corrections should be treated as stochastic parameters, implying that the user’s model needs to be extended and include unknown parameters for the ionospheric residuals, which one can weigh according to the distance of the user from the network receivers. In any other case, the position solutions will be biased even when the ambiguity success rate is high [36]. This model will be referred to hereafter as the ionosphere-weighted model, which was first introduced by [37].

Let us now assume that the network is able to provide, next to satellite clocks and satellite phase biases, regionally network-derived user-specific ionospheric corrections, the interpretation of which is based on Equation (7). In such a case, the uncombined terms of code and phase data will result in the following adapted formulation:

(10) $$\begin{array}{cc} E(\Delta \phi_{u,j}^{s}+{\hat{\tilde{dt}}}^{s}+\lambda_{j}{\hat{\tilde{\delta }}}_{,j}^{s}+\mu_{j}{\hat{\tilde{\iota }}}_{net\to u}^{\;s}) & =g_{u}^{s^{T}}\Delta x_{u}+d{\tilde{t}}_{u}+m_{u}^{s}\tau_{u}-\mu_{j}\left(\iota_{u}^{s}-\iota_{u,net\to u}^{s}\right)+\lambda_{j}\left({\tilde{\delta }}_{u,j}+{\tilde{a}}_{u,j}^{s}\right)\\ E(\Delta p_{u,j}^{s}+{\hat{\tilde{dt}}}^{s}-\mu_{j}{\hat{\tilde{\iota }}}_{net\to u}^{\;s}) & =g_{u}^{s^{T}}\Delta x_{u}+d{\tilde{t}}_{u}+m_{u}^{s}\tau_{u}+\mu_{j}\left(\iota_{u}^{s}-\iota_{u,net\to u}^{s}\right)+\mu_{j}{\tilde{d}}_{u,\mathrm{GF}} \end{array}$$

The interpretation of the estimable parameters in the ionosphere-weighted model is identical to the ionosphere-float counterpart, except for user’s phase biases that are biased by the network, instead of the user’s, DCB as shown in Table 2. The main difference between the two models is that the receiver code bias becomes estimable due to the introduction of the external ionospheric corrections. In our contribution we will show that the user DCB estimate lies at the meter level, which can degrade the positioning performance if ignored, as it has been observed in existing analysis [21].

The prevailing advantage of the ionosphere-weighted model becomes clear: one is able to a priori weigh the ionospheric residuals, $\iota_{u}^{s}-\iota_{net\to u}^{s}$, according to the ionospheric prediction error that depends on the network density, i.e., the user’s proximity to the network receivers, and improve the performance by this model strengthening. Introducing a priori stochastic pseudo-observables, as shown in Equation (11), extends our functional model of Equation (10), which gets a redundancy gain of m−1 at a single epoch compared to the ionosphere-float model.

(11) $$E\left(\Delta \iota \right)=\iota_{u}^{s}-\iota_{net\to u}^{s}$$

The provided ionospheric corrections will now be correlated both between themselves and in time, since they come from a previous adjustment. In this study, we neglect such correlation due to the excessive information that needs to be transmitted to the user and we weigh the ionospheric residuals based on the accuracy of the user-interpolated ionospheric corrections. This is empirically assessed by comparing the per-network-derived user-specific predicted corrections with the slant ionospheric delays at the user stations which have been estimated with ionosphere-float PPP-RTK and considered as truth.

In this case, the stochastic model is extended, as shown in Equation (12), assuming that all ionospheric pseudo-observations are assigned with the same a priori standard deviation $\sigma_{\Delta \iota }$ (that can be transmitted in real-time along with the corrections), neglecting any dependency on other factors (e.g., elevation angle):

(12) $$Q_{yy}=\mathrm{blkdiag}\left(Q_{\Delta {\tilde{\phi }}_{u}\Delta {\tilde{\phi }}_{u}},Q_{\Delta {\tilde{p}}_{u}\Delta {\tilde{p}}_{u}},Q_{\Delta \iota \Delta \iota }\right),\;\mathrm{with}\;Q_{\Delta \iota \Delta \iota }=\sigma_{\Delta \iota }^{2}\;I_{m}$$

## 3. Results and Analysis

In this section, we first introduce the data and processing strategy followed at both network and user components. We then present and analyze the network-corrections, focusing on the user-specific ionospheric corrections. In the following, we numerically demonstrate and analyze the performance of the single-system ionosphere-weighted PPP-RTK user using ionospheric corrections from multi-scale regional network configurations.

### 3.1. Data and Processing Strategy

**Figure 2:**
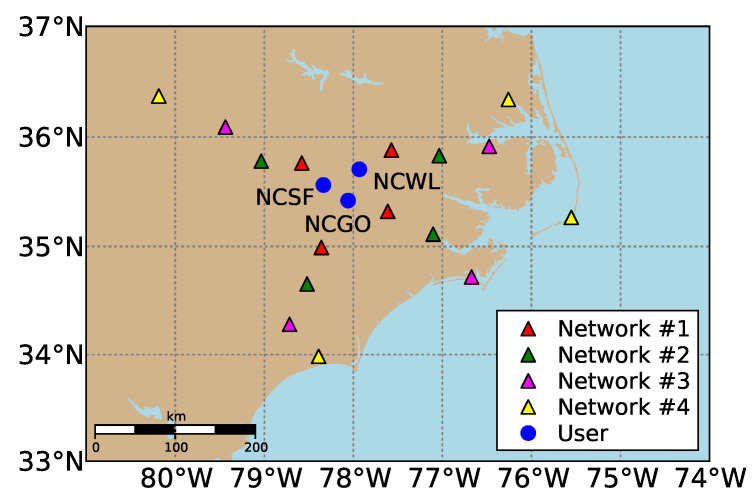
Geographic distribution of the selected CORS receivers in North Carolina used for the PPP-RTK network and user processing. The network receivers are classified in groups of four to form networks of varying inter-station distance and are denoted by red, green, magenta and yellow triangles in ascending order by distance. The remaining three receivers, denoted by blue dots, represent the user stations.

To carry out our case study, we used 24-h GPS dual-frequency code and phase data sampled every 30 s on 16 February 2014 (47th day of year), close to the solar maximum, from mid-latitude CORS receivers of the National Geodetic Survey (NGS) network in North Carolina, United States. Their geographic distribution is shown in Figure 2. To measure the impact of a network’s dimension on the achieved performance, we selected and split the network receivers into four evenly distributed networks; each consisted of four receivers, with average inter-station distances (user-to-reference receiver distances) of 68, 115, 174 and 237 km. The users are denoted by blue dots and are within the coverage of all four networks. During the selected day, there was a medium ionospheric disturbance since the final Kp-index ranged from 2o to 5o with a mean equal to 3+, as determined by GeoForschungsZentrum [38]. The final Kp-index is expressed in a scale of thirds and ranges between 0o, 0+, 1−, 1o, 1+ … all the way up to 9o (28 values in total).

At the network side, the GPS observations were processed independently for each network. In this study, we employed the geometry-plus-satellite-clock-fixed variant of the uncombined PPP-RTK network model [24] using IGS precise orbits and clocks, while the station coordinates were a priori precisely known. For the parameter estimation, the Kalman filter was utilized assuming that the receiver clok offsets and the slant ionospheric delays are unlinked in time. The carrier-phase ambiguities were treated as time-invariant parameters unless a cycle slip occurs, and the receiver and satellite phase biases were assumed to be time-constant as well. A random-walk stochastic process was assumed for the wet zenith tropospheric delay using a process noise of 0.1 $\mathrm{mm}/\sqrt{30s}$.

At the user component, the GPS observations were processed on a receiver-by-receiver basis for all user stations after being corrected by the IGS precise orbits and clocks and the network-derived satellite phase biases. The time correlation that was inherent in the latter products, as they come from the previous network adjustment, was neglected. Moreover, we started the user processing 1 h after the network processing had been initialized in order to allow the corrections to gain high precision, since in real conditions the network processing is assumed to generate corrections continuously. It is also important to note that the newly tracked satellites were excluded during their first few minutes at the user processing as the associated network corrections were not precise enough. The dynamic model settings for the Kalman-filtered PPP-RTK user processing were set identical to the network counterparts, with the only difference that the newly introduced parameters for the unknown receiver positions were assumed to be unlinked in time as we considered only kinematic positioning in this study. In case of the ionosphere-weighted user model, the receiver DCB was treated as a time-constant parameter. Although not shown in this contribution, we did not find any difference in the ambiguity resolution and positioning performance by treating the receiver DCB unlinked in time. It has been reported by Zhang and Teunissen [39] and Zha et al. [40] that receiver hardware temperature variations cause the receiver DCB to vary over time, which sould be taken into account in such cases.

At both the network and user levels, the uncombined code and phase measurements were empirically assigned with a zenith-referenced standard deviation of 30 cm and 3 mm, respectively, which is a reasonable choice for most applications [41], and were further weighted according to the sine of their elevation. A cut-off elevation angle of 10° was used to discard noisy measurements at low elevations. We assumed that no correlation existed between frequencies, as well as between code and phase measurements. In case of any tracked C1 observables from the receivers, they were aligned to the P1 observables using the monthly P1-C1 satellite DCB products provided by the Centre for Orbit Determination in Europe (CODE) in order to be consistent with the satellite clocks provided by IGS. Both PPP-RTK network and user data were corrected for a priori corrections, including tidal effects, phase windup and tropospheric delays. It is worth mentioning that the data in both components were processed in emulated real-time mode, since only forward filter processing was used.

We performed full (FAR) and partial (PAR) integer ambiguity resolution [42] with the LAMBDA (Least-squares AMBiguity Decorrelation Adjustment) method [15,43] using as input the float ambiguity solution, which was obtained from our Kalman filter in real-time based on our mixed-integer GNSS user model. It is important to notice that, unlike many studies, we did not a priori form any linear combinations of the ambiguities, such as the widelane combination, aiming to accelerate the search process. This is because the Z-transformation, embedded in the LAMBDA method, is known to maximally decorrelate the ambiguities by determining the optimal ambiguity combinations that transform the ellipsoidal ambiguity search space into more spheroid-like [44].

Further, we used the Fixed Failure-rate Ratio Test (FFRT) to decide whether or not the resolved ambiguities can be accepted as the *correct* ones [45], as an incorrect integer solution will hamper the positioning solutions. In this regard, a model-driven critical value was used with a fixed failure rate of 0.1% in order to have high confidence in the correctness of the integer outcomes. After the integer ambiguities had been accepted, we performed a single-epoch standard least-squares adjustment to obtain the ambiguity-resolved solution of the other parameters. The process of IAR+FFRT was executed on an epoch-by-epoch basis.

### 3.2. PPP-RTK Network Corrections

**Figure 3:**
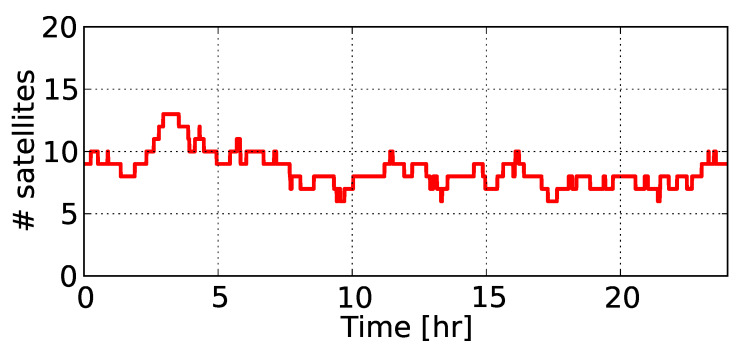
Number of GPS satellites tracked in network #1 during 16 February 2014.

In this section, we present the network-derived results. Figure 3 depicts the total number of commonly tracked satellites from at least two receivers in network #1 above the elevation cut-off angle of 10° during 16 February 2014. It can be seen that the number of commonly tracked GPS satellites varied from 6 to 13.

**Figure 4:**
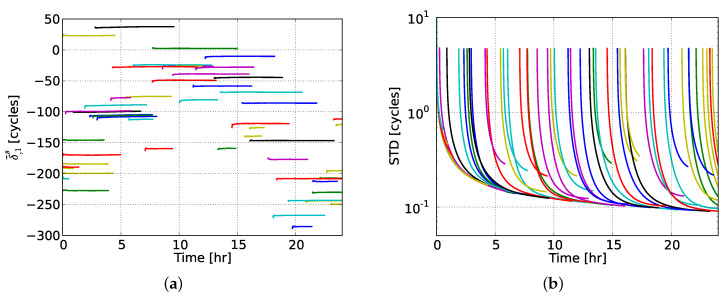
(**a**) Satellite phase bias estimates on L1 (in cycles) and (**b**) their formal standard deviations for all GPS satellites during the selected day. Each color represents a different GPS satellite.

Regarding the PPP-RTK network-derived estimates, we restricted our attention to the satellite phase biases and user-predicted ionospheric corrections. Figure 4 shows the satellite phase bias estimates on L1, ${\tilde{\delta }}_{,1}^{s}$, the interpretation of which can be seen from Table 1, along with their formal standard deviations (STDs) as determined from their vc-matrix. We chose to present the estimates of all GPS satellites in order to get a general insight into their behavior. It can be seen that the L1 satellite phase biases of the majority of GPS satellites showed remarkable stability over time. Most of these estimates achieved a formal precision of 0.20 cycles after 1–2 h, while the 0.10 cycles level was reached after 3–4 h. The longer a satellitewas observed from the network, the better the precision of its associated phase biases became over time.

The second set of PPP-RTK corrections we discuss here is the one of the user-predicted ionospheric corrections. After using the Kalman-filter-based slant ionospheric delay estimates of the network receivers, the undifferenced ionospheric corrections at the users were predicted on an epoch-by-epoch basis with the BLUP model, as discussed in Section 2.3. To make use of the ionosphere-weighted model at the PPP-RTK user processing, as shown in Equations (10)–(12), one has to make assumptions on the standard deviation of these corrections. To this end, the accuracy of the user-interpolated ionospheric corrections was assessed by comparing them to the estimated slant delays from an ionosphere-float PPP-RTK user processing. In such a comparison, one would get:

(13) $${\tilde{\iota }}_{u}^{s}-{\tilde{\iota }}_{net\to u}^{\;s}=\left(\iota_{u}^{s}-\iota_{net\to u}^{s}\right)+\left(d_{u,\mathrm{GF}}-d_{p^{\prime },\mathrm{GF}}\right)$$

Therefore, although one would expect their difference to be unbiased in the absence of ionospheric residuals, we recall that the user-predicted and user-estimated slant ionospheric delays differ by an unknown offset, that is the difference of the user and network-receiver DCBs. As already discussed, this network-user DCB needs to be estimated by the user.

**Figure 5:**
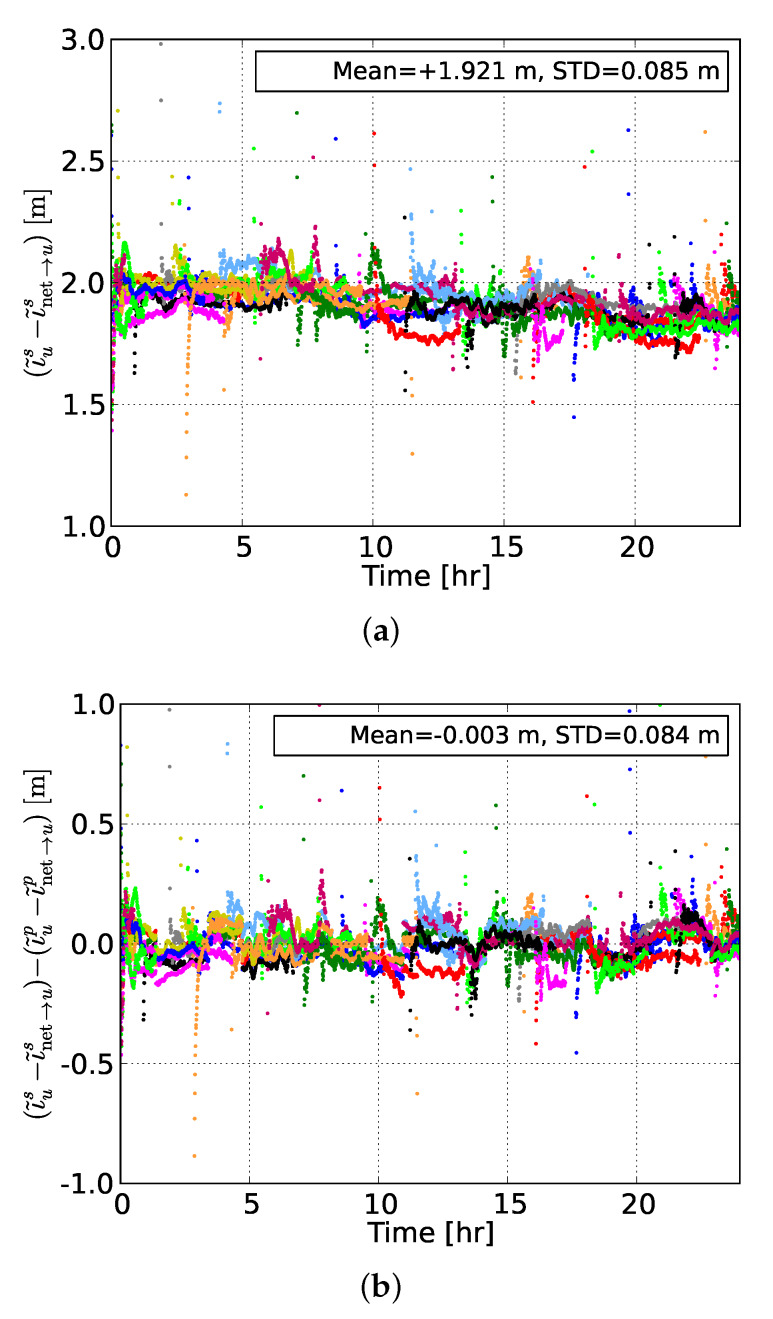
(**a**) Differences (in meters) of the undifferenced regionally network-derived user-predicted slant ionospheric delays and their counterparts estimated by the user NCWL, and (**b**) their between-satellite single-differenced results. Each color represents a different GPS satellite. This ionospheric correction prediction is referred to network #2 (mean station spacing of 115 km). The empirical mean and STDs were calculated for the complete 24-h time-series.

Figure 5 shows the differences between the user-estimated and user-predicted slant ionospheric delays, based on network #2, of all GPS satellites at the user station NCWL as well as their between-satellite single-differenced counterparts. It can be seen from the top panel that the time-series of differences between the estimated and predicted delays were biased by an offset of about 1.9 m. This is due to the remaining network-user DCB and it will be shown later that this is the value that the network-user DCB estimate fluctuates around. This bias was eliminated after applying a between-satellite single-differencing operator, with the resulting zero-mean time-series of differences being shown in the bottom panel of the same figure.

Using the ambiguity-fixed slant delays from the network receivers, the same procedure was followed for all *l* user stations to assess the accuracy of the ionospheric corrections per network:

(14) $$\sigma_{\Delta \iota }=\sqrt{\frac{\sum_{u=1}^{l}\sum_{i=1}^{k}\sum_{s=1}^{m}{\left(\Delta \iota_{u,i}^{s}-\Delta {\bar{\iota }}_{u}\right)}^{2}}{l\cdot k\cdot m-1}}$$

where $\Delta \iota_{u,i}^{s}$ denotes the ionospheric residual of the user *u* and satellite *s* at epoch *i*, $\Delta {\bar{\iota }}_{u}$ the average of the user-specific residuals (translated into the user DCB) over all satellites *m* and all epochs *k*.

### 3.3. Real-Time PPP-RTK Performance

The GPS dual-frequency data of the user stations were processed in kinematic mode, i.e., treating the receiver position as time-unlinked parameter, after being corrected for the network-derived estimates for satellite phase biases and predicted ionospheric corrections. The processing was performed with and without IAR, leading to ambiguity-float (PPP) and ambiguity-fixed (PPP-RTK) results, considering both the ionosphere-float and ionosphere-weighted models.

**Figure 6:**
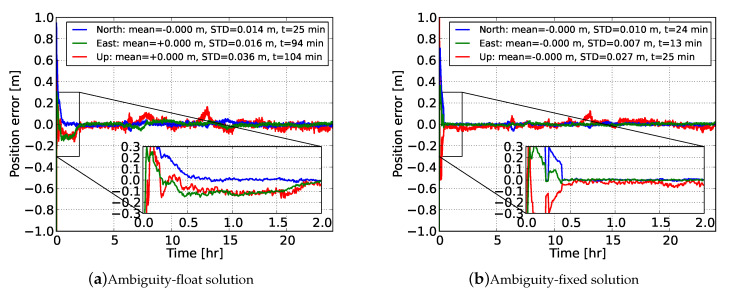
Time-series of the GPS dual-frequency ionosphere-float (**a**) PPP and (**b**) Full integer ambiguity resolution (FAR)-based PPP-RTK kinematic user position for station NCWL with respect to its ground-truth. The empirical means and STDs are calculated for the estimated positions after 2 h. A zoom-in window during the first 2 h is provided.

To get an initial numerical insight into the achieved positioning performance, we commence our discussion with Figure 6, which illustrates the ambiguity-float and ambiguity-resolved kinematic user position solutions with respect to the ground truth for the arbitrarily chosen station NCWL. The convergence time was defined here as the minimum accumulated observational time span required to achieve accuracy (position error with respect to ground truth) better than 10 cm for the remaining time window. One can observe that the ionosphere-float PPP solution achieved cm-level accuracy during the 24-h period. It was seen, though, that a long convergence time was needed to reach the 10 cm level, namely 25, 94 and 104 min for the north, east and up components, respectively. It can be seen that at about 13 h the error along the up component deviated instantly from the 10 cm level, which is probably due to errors contaminated in the measurements that were not filtered out in our user processing.

The performance gain via single-receiver PPP-RTK ambiguity resolution is shown in Figure 6b. The benefit in terms of precision is evident at the beginning of the time-series, while this is not the case after a few hours. This is due to the fact that the float ambiguities get more precise over time and as a result the positioning solution’s precision is dictated by the carrier-phase measurements. In the ambiguity-fixed case, the convergence time was reduced to 24, 13 and 25 min along the north, east and up components, respectively, while the accuracy reached the level of 1.0, 0.7 and 2.7 cm, respectively.

It is interesting to note that the north error got better than 10 cm at the 13th min (same as the east error) but deteriorated in the subsequent epochs. A consistent accuracy better than 10 cm along the north component was achieved from the 24th min onwards. This was due to the inclusion of a new satellite above the elevation cut-off angle in the solution. In the above ambiguity-resolved solutions, the process of FAR and FFRT was repeated at every epoch with the fixed ambiguity (and updated position) solution being accepted only when the FFRT was passed. This is as expected, since the float solution needed time to converge, implying that each time new satellites were tracked it would take a few epochs before the ambiguities could be reliably fixed.

**Figure 7:**
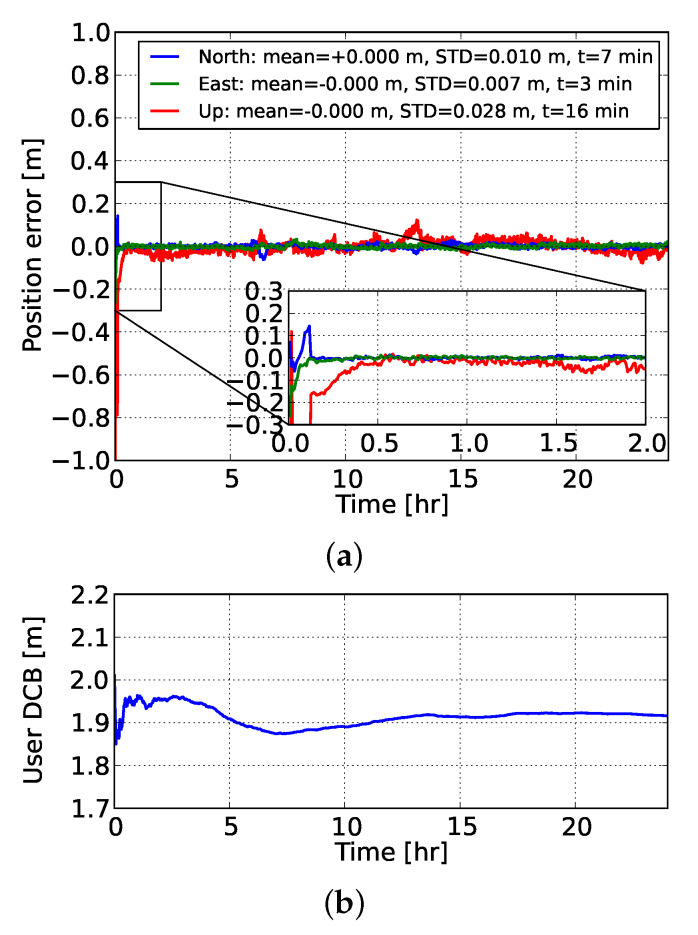
Time-series of (**a**) the GPS dual-frequency ionosphere-weighted FAR-based PPP-RTK kinematic user position for station NCWL with respect to its ground-truth, and (**b**) its associated network-user scaled DCB estimate. The ionospheric corrections were determined from network #2. The empirical means and STDs are calculated for the estimated positions after 2 h. A zoom-in window during the first 2 h is provided.

Then, as our initial goal was to evaluate the gain in PPP-RTK user positioning performance by using precise ionospheric corrections, we used the network-derived predictions to strengthen the ionosphere-float user model, turning it into an ionosphere-weighted one. Figure 7 shows the time-series of the ionosphere-corrected PPP-RTK user kinematic position errors using corrections from network #2. It is evident that the a priori ionospheric information played a substantial role in reducing the convergence time, since it took only 7 and 3 min for the north and east ambiguity-fixed position errors to converge below 10 cm. This was more than 3 times faster compared to the ionosphere-float model. The estimable user code bias is shown in the same figure. It can be seen that the user DCB estimate showed a stable temporal behavior and, more importantly, it fluctuated around the mean value of the differences between the user-predicted and user-estimated ionospheric delays, as shown in Figure 5.

#### 3.3.1. Convergence Time

Although the provision of ionospheric corrections seems to bring a substantial improvement in convergence time, a single solution cannot be assumed to be representative of the general case. Due to the random nature of the GNSS data, a large number of samples are required to infer the empirical distribution of the achieved convergence times and to come up with realistic deductions. To that end, we processed the data of all user stations using both FAR and PAR on 16 February 2014, with a 3-h processing window being re-initialized every 1 min, in order to capture the different receiver-satellite geometry changes and obtain a representative sample of solutions (3780). The computed absolute horizontal (radial) position errors with respect to the ground-truth were collected and sorted for each epoch according to their magnitude. Further, we identified the 50th and 90th percentiles of the 2D horizontal errors, and obtained the so-called percentile curves as a measure to represent the convergence times.

**Figure 8:**
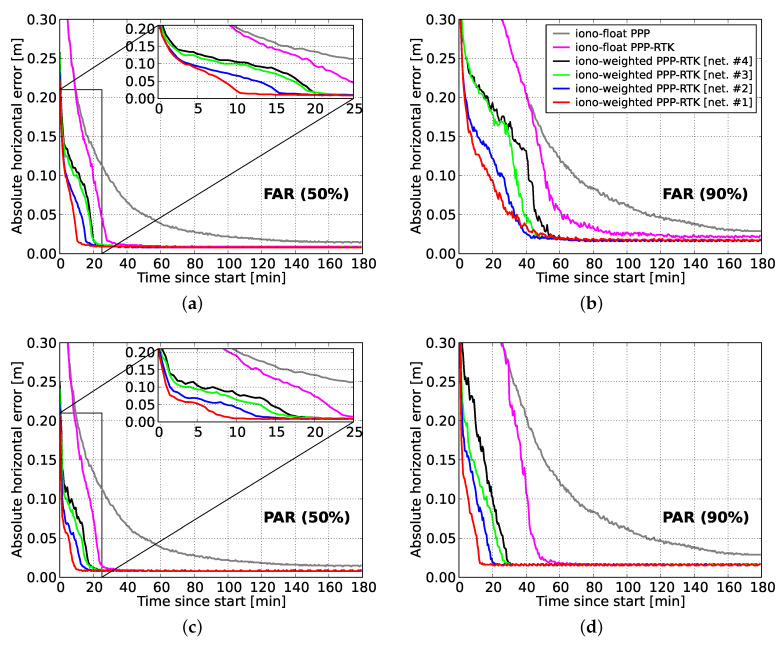
Convergence behavior of the horizontal radial positioning errors for (**a**) 50% of the FAR-based solutions, (**b**) 90% of the FAR-based solutions, (**c**) 50% of the PAR-based solutions, and (**d**) 90% of the PAR-based solutions of all user stations as a function of time since the processing start. The processing window has been re-initialized every 1 min within the selected day for all available solutions and networks.

The convergence behavior of the user positioning results with and without IAR, as well as by utilizing predicted ionospheric corrections from multi-scale networks are discussed in the following. Figure 8 shows the 50th and 90th percentiles of the absolute horizontal errors for the first 3 processing hours. The ambiguity-float results show that 28.5 and 68.5 min are needed to let 50% and 90% of the horizontal position errors to converge, respectively. The gain due to single-receiver IAR is evident in both FAR- and PAR-based results, as for 90% of the samples the convergence times reduced to 51.0 (26% improvement) and 41.0 (40% improvement) min, respectively. This shows that single-receiver PPP-RTK ambiguity resolution could reduce the convergence time substantially, since the convergence curves had a sharper decrease, especially with PAR. Similar results were reported by Odijk et al. [13] and Zhang et al. [24], in the context of ionosphere-float ambiguity-fixed kinematic positioning results, with an about 50 and 45 min convergence time, respectively.

**Figure 9:**
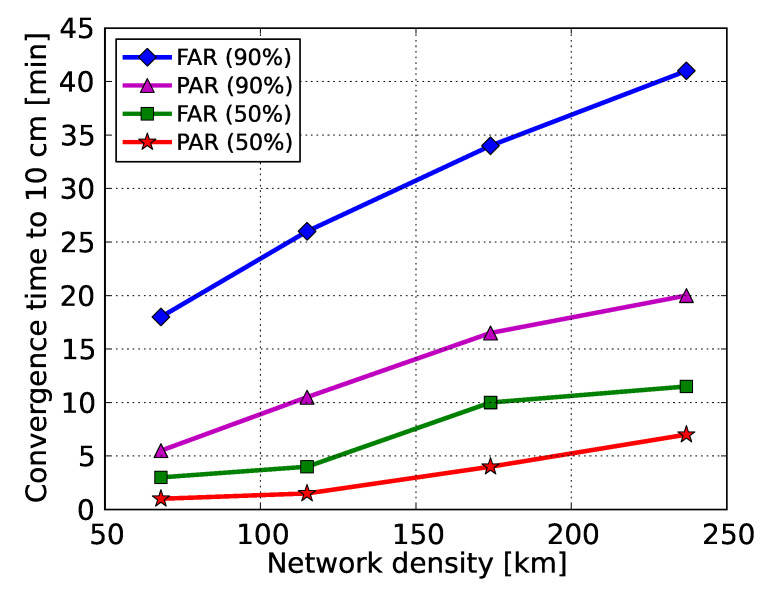
Convergence time of the horizontal radial position errors to 10 cm as a function of the network density for both FAR and PAR, based on 50% and 90% of the sample solutions.

In this study, we present to our knowledge for the first time percentile convergence curves when precise ionospheric corrections are employed from multi-scale regional networks. From Figure 8, it can be seen that the time required for the FAR-based horizontal position errors to get below the decimeter level reduced to 41 to 18 min (90% of samples) for network spacings between 237 and 68 km thanks to the use of the ionospheric corrections. As expected, the smallest-scale network #1 with a mean station spacing of 68 km provided the best performance, while the largest-scale network #4 with a mean station spacing of 237 km gives the worst. However, the latter still provided better performance compared to the ionosphere-float PPP-RTK case where the slant ionospheric delays were entirely unknown. It is also interesting to notice that these convergence times showed a linear relationship with the average inter-station spacing, as shown in Figure 9, which demonstrates the clear impact that the dimension of a network had on the achieved user’s performance.

Further reduction in the convergence times can be seen from the PAR-based results. In particular, the 90% percentile curves show that the time needed to surpass the decimeter level ranged from 5.5 to 20.0 min for network spacings between 68 and 237 km, showing the superior performance of PAR over FAR. This is due to the fact that regional ionospheric corrections were able to strengthen the underlying model in such a way that a large enough subset of ambiguities could be identified and fixed in a shorter time span to allow for centimeter-level position results. It is also remarkable that for 50% of the cases the regional corrections were able to reduce the convergence times to 1.0, 1.5, 4.0 and 7.0 min for the 68, 115, 174 and 237 km spaced networks, respectively. The linear relationship between the convergence times and the average inter-station spacing is also obvious in PAR for both 50% and 90%, as shown in Figure 9. Therefore, we conclude that the GPS-only ionosphere-weighted PAR-based PPP-RTK user convergence times to 10 cm can be less than 6 min when regional ionospheric corrections from a 68 km spaced network are used in 90% of the cases. For 50% of the cases this even reduces to 1 min. These results are valid for a medium ionosphere-disturbed day. In the case that corrections from an about 237 km spaced network are used, the convergence times are expected to be shorter than 20 min in a single-system dual-frequency solution.

#### 3.3.2. Positioning Accuracy

**Figure 10:**
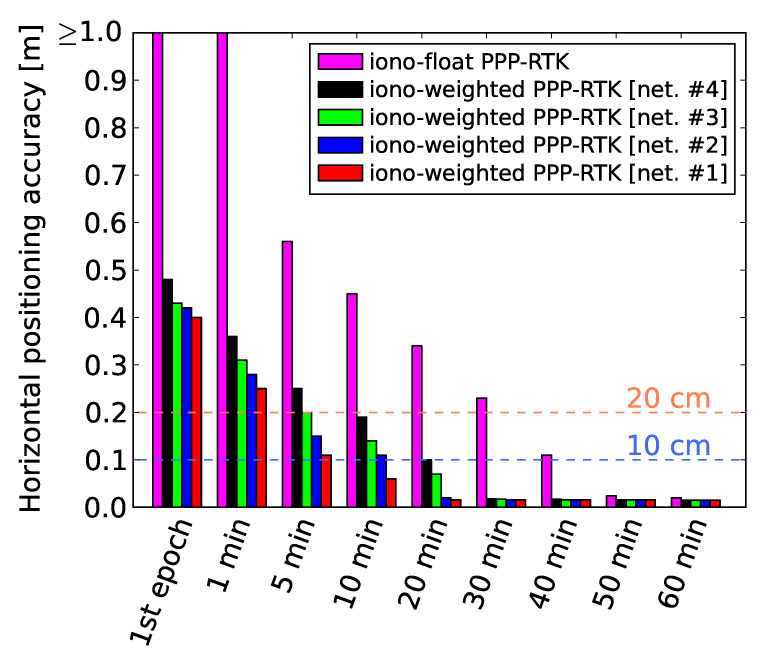
Horizontal positioning accuracy (90th percentile) at the first epoch and 1, 5, 10, 20, 30, 40, 50, 60 min since start for the PAR-based PPP-RTK user solutions.

It is also of interest to get an insight into the achieved partially-ambiguity-fixed PPP-RTK user positioning accuracy using external ionospheric corrections. Figure 10 shows the 2D horizontal positioning errors at the first epoch and several minutes since start based on 90% of the sample runs. It can be observed that centimeter-level accuracy could be achieved when a long time span was accumulated in the filter. Although the errors were at the meter-level at the first minute, the regional ionospheric constraints could improve the accuracy by more than 60%, with the accuracy reaching the 25 cm level when corrections from network #1 were used. The differences in the achieved accuracy using ionospheric information from multi-scale networks were evident, which were absent when the time span was longer than 30 min as the underlying model was strong enough to allow for PAR-based cm-level accuracy. It is important to notice here that 20 cm accuracy could be achieved within the first 5 min of processing using a four-receiver network of maximum 174 km spacing, while at the same time the accuracy could be even 11 cm if a 68 km spaced network was used. Using data over a time span of 20 min showed that all networks used in this study were able to provide ionospheric corrections that lead to sub-10 cm horizontal accuracy, while the smallest-scale network can bring the accuracy down to 1.5 cm. If the time span was longer, then it can be seen that the performance was equivalent for all networks. One concludes, therefore, that there is a significant improvement at the first processing epochs by using regional ionospheric corrections, with the performance being linearly scaled based on the network density.

Similar performance should be expected from users when they perform PPP-RTK positioning in similar conditions as those of the current study. The convergence times may vary depending on the user’s geographical location, atmospheric activity, receiver type and possible multipath contamination due to the nearby environment. It has to be noted, though, that such results should not be expected in equatorial areas and during ionospheric storms.

## 4. Conclusions

In this contribution, we rigorously analyzed the key role of ionospheric corrections in achieving fast high-precision positioning. To this end, we measured the impact that the network density has on the achieved performance, for the first time in terms of PPP-RTK. Given that the data-driven integer-estimable ambiguities have been successfully mapped to their correct integers, the observational time span required to reach high positioning accuracy can be greatly reduced compared to PPP. In case there is no a priori information about the ionosphere, the PPP-RTK user model is weak in terms of its ambiguity resolution capabilities because the unknown parameters for the ionosphere need to be estimated.

We first gave a detailed presentation of the uncombined PPP-RTK network and user models, along with their parameter estimability and interpretation. The transition from the ionosphere-float to the ionosphere-weighted variant of the PPP-RTK user model is achieved through incorporating prior information about the ionosphere. It was shown that such user-specific ionospheric corrections can be predicted with BLUP based on the network-derived slant delays and then used at the user to achieve fast PPP-RTK ambiguity resolution and, therefore, fast convergence.

We also numerically demonstrated for the first time the capabilities, in terms of positioning convergence curves and achieved accuracy, of the GPS-only dual-frequency ionosphere-weighted PPP-RTK user model using ionospheric corrections from multi-scale regional networks. To evaluate the effect that the network stations’ spacing has on the user performance through the ionospheric corrections, we processed GPS dual-frequency data from four regional networks with varying station spacings, ranging from 68 to 237 km. We determined and presented the PPP-RTK network results, including the stable satellite phase biases over time and the predicted user-specific slant ionospheric delays, as well as their quality.

Given the network corrections, ground-truth coordinates and datasets from several single-receiver users, we computed a large number of kinematic 2D horizontal positioning error samples to get representative convergence curves. As numerically shown, for 90% of the samples the convergence times of the ambiguity-float solutions to reach 10 cm were reduced from 68.5 to 51.0 min after full ambiguity resolution and to 41.0 min after partial ambiguity resolution. It was then demonstrated that when ionospheric corrections are available from the smallest-scale network of 68 km spacing, sub-10 cm horizontal accuracy can be achieved almost instantaneously in the ionosphere-weighted partially-ambiguity-fixed solutions, with 90% of them requiring less than 6 min. Moreover, it was empirically found that the convergence time bears a linear relationship with the mean inter-station distance of the considered networks, with the smallest one providing the best performance, as expected. Based on the 50th (90th) percentile of 2D horizontal positioning errors, sub-decimeter level accuracy can be reached within 1.5 (10.5), 4.0 (16.5) and 7.0 (20.0) min when ionospheric corrections from networks of 115, 174 and 237 km spacing are used, respectively, showing that sparser networks can provide sufficiently precise ionospheric information to achieve faster PPP-RTK solutions.

Based on the above performance studies, further improvement in the kinematic PPP-RTK convergence time can be expected when multi-frequency and/or multi-GNSS data are integrated, as it has been shown for the ionosphere-float model (see e.g., [18,46]). We, therefore, believe that the synergetic use of multiple systems and frequencies will further improve the ionosphere-weighted single-system PPP-RTK user performance, which we will investigate in the future.

## Figures and Tables

**Table 1:** Estimable dual-frequency PPP-RTK network parameters and their interpretation using the Common Clocks S-system (the symbol *p* denotes the pivot satellite/receiver if it is used as superscript/subscript).

Estimable Parameter	Interpretation
Receiver clock	$d{\tilde{t}}_{r\neq p}=dt_{pr}+d_{pr,\mathrm{IF}}$
Satellite clock	$d{\tilde{t}}^{s}=\left(dt^{s}+d_{,\mathrm{IF}}^{s}\right)-\left(dt_{p}+d_{p,\mathrm{IF}}\right)$
Ionospheric slant delay	${\tilde{\iota }}_{r}^{s}=\iota_{r}^{s}+d_{r,\mathrm{GF}}-d_{,\mathrm{GF}}^{s}$
Receiver phase bias	${\tilde{\delta }}_{r\neq p,j}=\delta_{pr,j}-\frac{1}{\lambda_{j}}\left(d_{pr,\mathrm{IF}}-\mu_{j}d_{pr,\mathrm{GF}}\right)+a_{pr,j}^{p}$
Satellite phase bias	${\tilde{\delta }}_{,j}^{s}=\delta_{,j}^{s}-\frac{1}{\lambda_{j}}\left(\left[d_{,\mathrm{IF}}^{s}-d_{p,\mathrm{IF}}\right]-\mu_{j}\left[d_{,\mathrm{GF}}^{s}-d_{p,\mathrm{GF}}\right]\right)-\delta_{p,j}-a_{p,j}^{s}$
Phase ambiguity	${\tilde{a}}_{r\neq p,j}^{s\neq p}=a_{pr,j}^{s}-a_{pr,j}^{p}$

Note: ${\left(\cdot \right)}_{,\mathrm{IF}}=\frac{1}{\mu_{2}-\mu_{1}}[\mu_{2}{\left(\cdot \right)}_{,1}-\mu_{1}{\left(\cdot \right)}_{,2}]$; ${\left(\cdot \right)}_{,\mathrm{GF}}=-\frac{1}{\mu_{2}-\mu_{1}}[{\left(\cdot \right)}_{,1}-{\left(\cdot \right)}_{,2}]$; ${\left(\cdot \right)}_{ij}={\left(\cdot \right)}_{j}-{\left(\cdot \right)}_{i}$

**Table 2:** Changes in parameter estimability and interpretation in the PPP-RTK user model due to the introduction of external ionospheric corrections.

Estimable Parameter	Interpretation
Receiver phase bias	${\tilde{\delta }}_{u,j}=\delta_{pu,j}-\frac{1}{\lambda_{j}}\left(d_{pu,\mathrm{IF}}-\mu_{j}d_{pp^{\prime },\mathrm{GF}}\right)+a_{pu,j}^{p}$
Receiver code bias	${\tilde{d}}_{u,\mathrm{GF}}=d_{u,\mathrm{GF}}-d_{p^{\prime },\mathrm{GF}}$
